# Rare fungal keratitis caused by plant pathogens: report of two cases and review of the literature

**DOI:** 10.3389/ffunb.2026.1785252

**Published:** 2026-03-25

**Authors:** Zixiang Zhao, Lijuan Xiang, Yu Liu, Shaoping Xu, Yang Chen, Man Yu

**Affiliations:** 1 Department of Ophthalmology, Sichuan Provincial People’s Hospital, School of Medicine, University of Electronic Science and Technology of China, Chengdu, China; 2 Department of Cardiovascular Medicine, Sichuan Provincial People’s Hospital, School of Medicine, University of Electronic Science and Technology of China, Chengdu, China

**Keywords:** *Macrophomina phaseolina*, *Colletotrichum fructicola*, fungal keratitis, metagenomic next-generation sequencing, diagnosis, review

## Abstract

*Macrophomina phaseolina* and *Colletotrichum fructicola* are notable plant pathogens, yet cases of keratitis from these fungi are rarely reported. Limited awareness of this keratitis etiology among ophthalmic professionals reduces the likelihood of accurate diagnosis and timely treatment. This report aims to improve the understanding of these rare infections in eye care. We present two cases of keratitis: one caused by *M. phaseolina* and another by *C. fructicola*, both of whom experienced a complicated treatment course. Traditional fungal exams yielded negative results, which limited disease identification and focused therapy. To determine the cause, we used metagenomic next-generation sequencing (mNGS) on clinical samples obtained from corneal scrapings. The mNGS report was received during therapy and quickly identified the pathogen. Based on this, we looked for treatment regimens for this kind of infection in previous literature, altered and implemented appropriate antifungal drug therapy, and the patient’s condition improved. We review the literature from 1970 to 2025 on *M. phaseolina* and *Colletotrichum* spp. keratitis. We identified 10 cases of *M. phaseolina* keratitis from four studies and 72 cases of *Colletotrichum* spp. keratitis, including five of *C. fructicola*, in 43 articles. Misdiagnosis was common due to limited clinical and microbiologic suspicion. The rise of infections by rare pathogens highlights diagnostic challenges. Traditional methods often delay accurate diagnosis, while mNGS enables rapid identification of pathogen, crucial for effective treatment and vision preservation.

## 1 Introduction

Fungal keratitis is a severe corneal infection that accounts for 30-40% of all cases of microbial keratitis. It is the second leading cause of blindness in developing countries (Whitcher et al., 2001). According to Brown et al., the annual global incidence of this disease is estimated to be 1,051,787 cases (736,251-1,367,323) (Brown et al., 2021). The disease predominantly affects tropical and subtropical regions with hot and humid climates, accounting for 20-60% of corneal infections in these areas (Bongomin et al., 2017). The prevalence of fungal infections has increased in recent years due to the irrational use of antibiotics and the increasing number of patients undergoing hormonal and immunosuppressive treatments (Benedict et al., 2019).

Fungal keratitis is a severe condition that often resists treatment and has a worse prognosis than other types of infectious keratitis (Ali Shah et al., 2017). Early and accurate diagnosis is crucial to determine the appropriate treatment and effectively eradicate infection (Chang and Chodosh, 2011). Fungal culture is the gold standard for diagnosis, but its prolonged culture time and low positive rate to atypical corneal fungal infections make it unsuitable for early diagnosis. Other diagnostic techniques, such as sampling microscopy, fungal staining, and *in vivo* confocal microscopy (IVCM), have a low positive rate for early detection of rare or unusual fungi (Badiee et al., 2010), which frequently delays diagnosis and potentially leads to vision loss or blindness. Early intervention in cases of fungal keratitis is crucial. When clinicians cannot identify pathogenic microorganisms, the mNGS of clinical samples should be performed immediately. This aids in the diagnosis and guides the clinical application of appropriate medication based on the results.

In this study, we describe two rare cases of fungal keratitis caused by plant pathogens and a systematic review of corneal infections caused by *Macrophomina phaseolina* and *Colletotrichum* spp. This research uses unique instances as the basis for a thorough examination of this clinical issue. We emphasize the crucial role of mNGS in detecting changes in pathogenicity dominance and in guiding the selection of therapeutic drugs for infectious keratitis.

## 2 Case reports

### 2.1 Case 1

**Figure 1:**
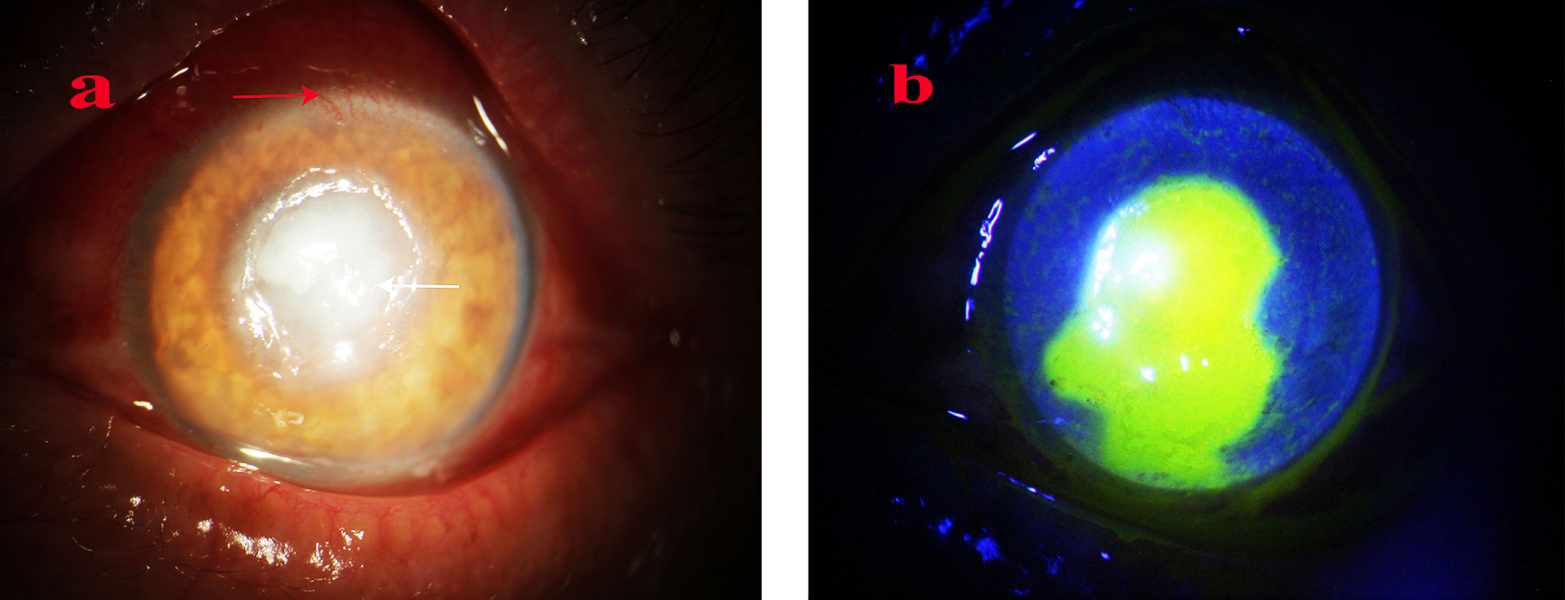
Anterior segment image of Case 1 at the initial visit. A substantial white abscess in the central corneal ulcer is notable (white arrow) and neovascularization in the anterior corneal limbus (red arrow). **(a, b)** Slit lamp and sodium fluorescein staining at the first visit, respectively.

**Figure 2:**
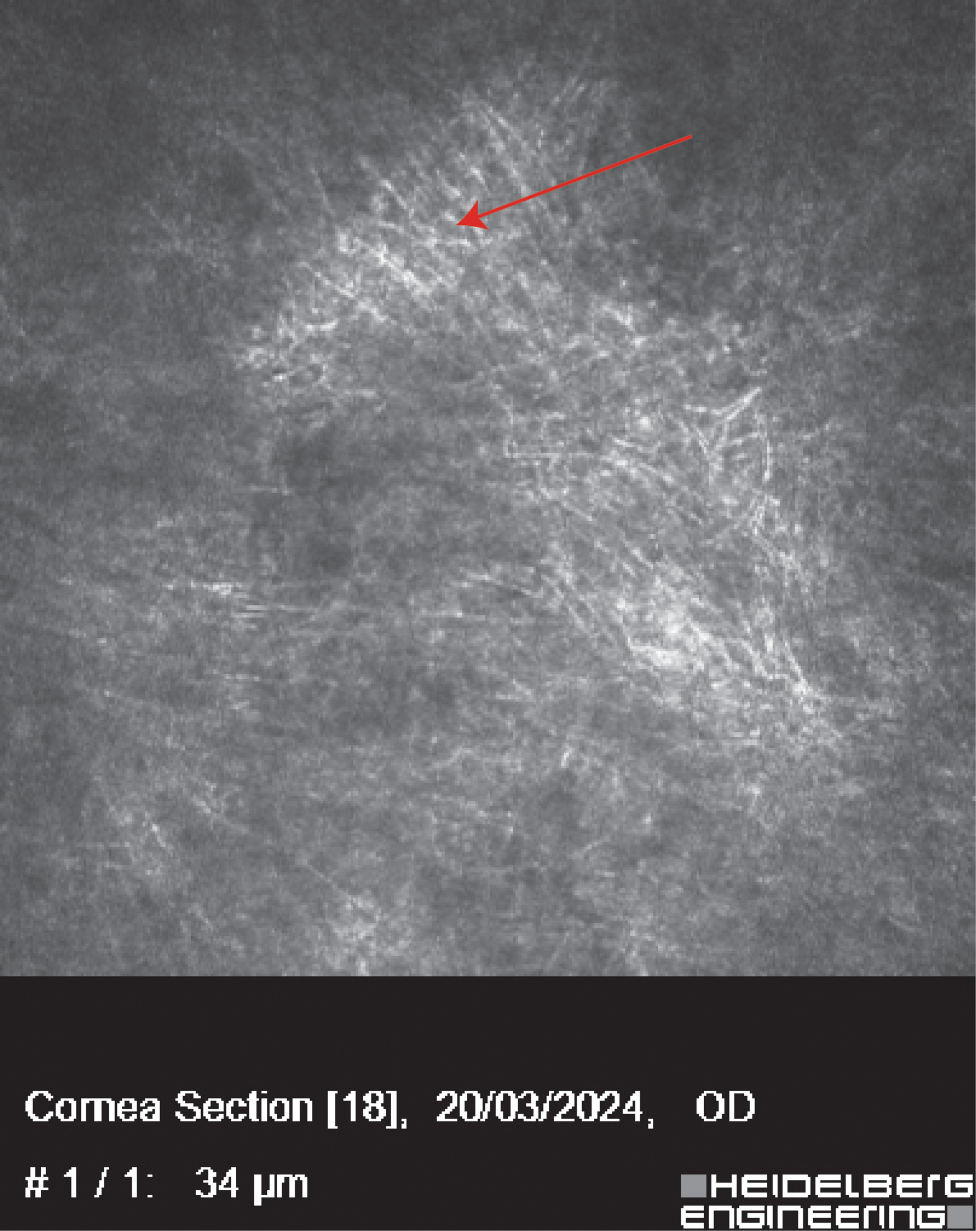
The IVCM of Case 1 showed massive hyperlinear structures in the stromal layer, which implies fungal infection.

A 75-year-old man was presented to the ophthalmology outpatient department with complaints of severe pain, redness, and tearing in his left eye. The patient had no history of diabetes, immunological disorders, or the use of immunosuppressive medications or other relevant drugs. The patient reported feeling a foreign body sensation in the left eye that began five months ago, without previous ocular trauma. He was diagnosed with keratitis in the left eye in a local hospital and treated with unspecified antifungal medications, antibiotics, and corticosteroids. However, his symptoms continue to recur. The patient was referred to our hospital due to a significant worsening of symptoms and substantial vision impairment. At admission, the best corrected visual acuity in the left eye was limited to hand movements only. Slit-lamp examination revealed conjunctival hyperemia and neovascularization that extended to the cornea from the superior and temporal corneal margins. Furthermore, a central corneal ulcer measuring 4mm × 4mm and corneal edema (**Figures 1a, b**). Microscopic examination of the patient’s corneal scrapes employing calcofluor white (CFW) fungal staining showed negative results. The IVCM image showed groups of hyperlinear structures in the stroma that were typical signs of fungal hyphae, as shown in **Figure 2**. Subsequently, the patient was diagnosed with fungal keratitis and treated hourly with 5% natamycin eye drops. To identify fungus species, the corneal scraping clinical sample was sent for mNGS analysis. An mNGS analysis identified the pathogen as *M. phaseolina* (GenBank accession numbers GCA_008729105.1), with 4 sequences with a relative abundance of 2.34%. Due to the negative results of the microbial culture, a drug sensitivity test could not be performed. Review of the literature indicated that *M. phaseolina* is susceptible to voriconazole, itraconazole, and amphotericin B based on several *in vitro* studies. It also exhibits moderate susceptibility to natamycin and posaconazole (Tan et al., 2008; Ahirwar et al., 2022), which is consistent with the treatment results in this case. Due to the inaccessibility of amphotericin B, the patient received topical administration of 1% voriconazole and 5% natamycin eye drops. Additionally, 0.1% voriconazole was injected into the stroma and conjunctiva surrounding the corneal ulcer. Given normal liver and renal function, intravenous voriconazole therapy was initiated at a dose of 200 mg twice daily. This therapeutic regimen resulted in significant clinical improvement. After 3 months, the lesion was completely epithelialization with a corneal scar.

### 2.2 Case 2

**Figure 3:**
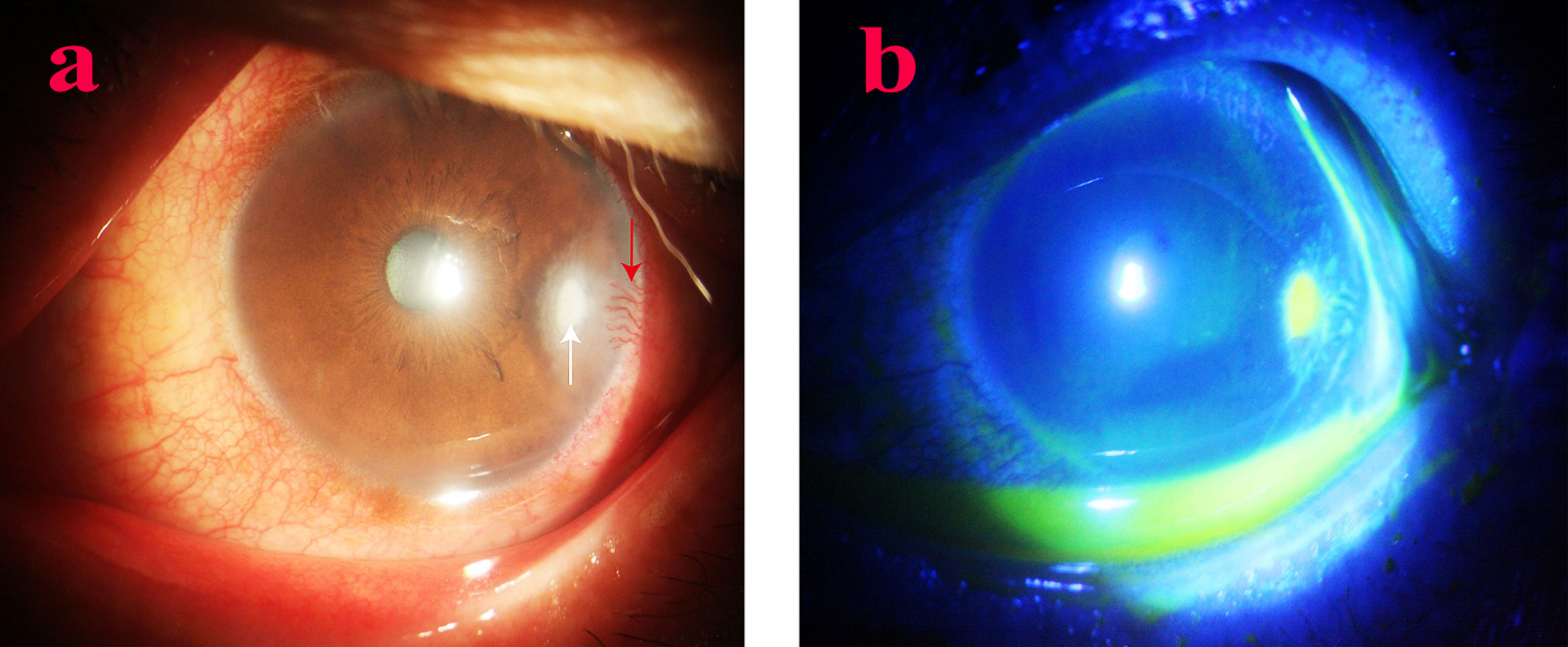
Anterior segment image of Case 2 at the initial visit. The white abscess in the temporal cornea is notable (white arrow), and neovascularization in the temporal corneal limbus (red arrow). **(a, b)** Slit lamp and sodium fluorescein staining at the first visit, respectively.

A 51-year-old male presented to the ophthalmology outpatient department with complaints of pain, redness, and tearing in his left eye. He had a two-year history of diabetes mellitus, with stable glycemic control maintained through regular pharmacologic management. There was no history of immunological disorders or use of immunosuppressive or other relevant medications. The patient complained that his symptoms began four months earlier when grass clippings entered his left eye. He was diagnosed with fungal keratitis in his left eye at a local hospital and prescribed oral itraconazole (400 mg/day), 0.2% fluconazole eye drops administered hourly, tobramycin eye drops administered hourly and intravenous clindamycin (300 mg/day). Subsequently, he was discharged from the hospital as his symptoms improved. However, the patient’s symptoms worsened, leading to a visit to our outpatient clinic. On initial examination, the best corrected visual acuity was 20/25. A slit-lamp examination of the left eye revealed conjunctival hyperemia, neovascularization of the temporal corneal limbus, a 1mm × 1mm ulcer in the temporal cornea, and mild corneal edema (**Figures 3a, b**). Microscopic examination of the patient’s corneal scrapes employing calcofluor white (CFW) fungal staining showed negative results. An mNGS analysis identified the pathogen as *C. fructicola* (GenBank accession numbers GCA_009771025.1), with 4207 sequences with a relative abundance of 99.72%. Given the patient’s refusal to receive IVCM and the prior identification of the pathogen via mNGS, IVCM was ultimately not administered. *C. fructicola* is affiliated with the *C. gloeosporioides* complex. Due to the negative results of the microbial culture, a drug sensitivity test could not be performed. However, research indicates that *C. gloeosporioides* is sensitive to triazole antifungal agents such as voriconazole, posaconazole and itraconazole, in addition to natamycin and amphotericin B (Takezawa et al., 2010; Lamarca et al., 2016; Wang et al., 2020; Izadi et al., 2021; Imai et al., 2022). Cao et al. reported that natamycin exhibited significant bacteriostatic activity against *C. fructicola* in infected apples (Cao et al., 2023). The patient received treatment with topical administration of 1% voriconazole and 5% natamycin eye drops every hour. With normal liver and renal function, we supplemented with 200 mg of oral itraconazole twice a day. After 1 month of treatment, the patient’s best corrected visual acuity improved to 20/20 and the lesion was completely epithelialized.

## 3 Discussion

Fungal keratitis is a serious ocular disease that can lead to blindness. Fungal keratitis predominantly affects people in developing countries within tropical and subtropical regions, where a significant portion of the population participates in agricultural activities. Due to the lack of diagnostic tools in these areas, the actual incidence of the disease can be much higher than estimated (Rose-Nussbaumer et al., 2016). The disease can be caused by more than 100 different fungal infections, but more than 95% of cases of fungal keratitis are caused by *Fusarium* spp. *Aspergillus* spp. and the yeast *Candida* spp (Thomas et al., 2005).

Fungal keratitis can cause severe corneal damage, requiring surgical intervention in up to 25% of cases. Despite surgery, nearly 60% of patients suffer from unilateral blindness. This outcome is primarily due to diagnostic delays, caused by the low positivity rate of conventional clinical diagnostic methods or prolonged diagnostic periods, which hinder timely and accurate treatment guidance (Burton et al., 2011).

*M. phaseolina* and *C. fructicola* are important plant pathogenic fungi, and their infections that result in fungal keratitis have rarely been documented (Natarajan et al., 2013; Lhoussni et al., 2014). Our article further elucidates the clinical and demographic characteristics of *M. phaseolina* and *C. fructicola* keratitis, emphasizing the importance of mNGS for the early diagnosis of fungal keratitis to guide clinical management.

### 3.1 Review of the literature

#### 3.1.1 Global literature review: *M. phaseolina* keratitis from 1970 to August 1, 2025

**Figure 4:**
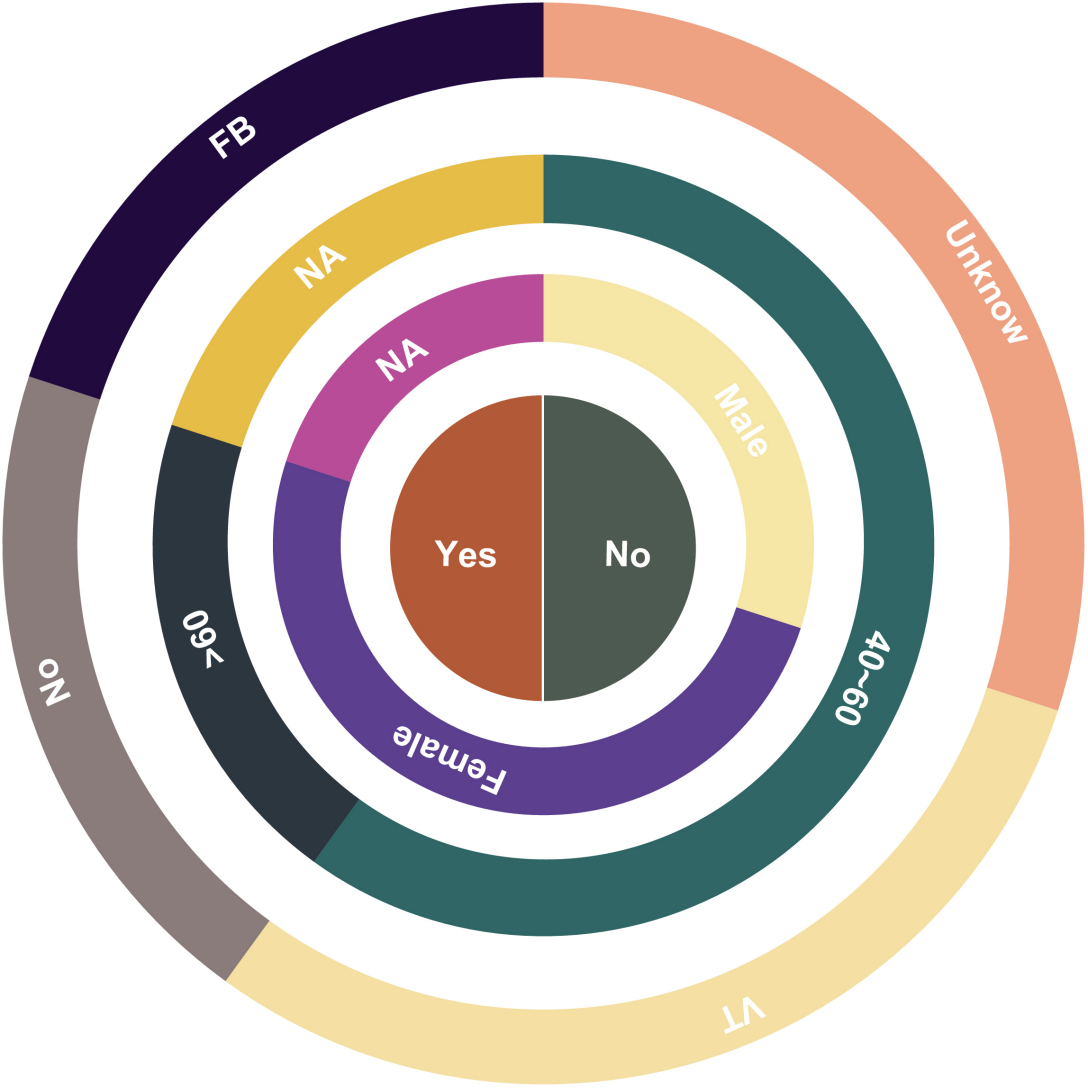
Statistical chart depicting the clinical and epidemiological characteristics of corneal infections caused by *Macrophomina phaseolina*. Risk factors, age, sex, and therapeutic keratoplasty are shown in order from the outer ring to the inner ring. (NA, not available; FB, foreign body; VT, vegetative trauma).

A systematic search for FK cases caused by *M. phaseolina* from 1970 to August 1, 2025, was performed on PubMed, Embase, and Medline search engines, using the keywords ‘*Macrophomina phaseolina*’, ‘Case Reports’, and ‘Infections’. Additionally, we performed a manual search of the reference lists of included studies using a snowball technique to identify further relevant articles. Seven publications about *M. phaseolina* infection were ultimately included, four describing corneal infections. The search revealed only 13 cases of all infections reported due to *M. phaseolina* in the world in the last five decades or so. Of these, 10 cases were associated with KF (Bagyalakshmi et al., 2008; Premamalini et al., 2012; Ahirwar et al., 2022; Tandra et al., 2025). Other clinical manifestations included cutaneous infections (n=2) (Srinivasan et al., 2009; Schwartz and Kapila, 2020) and disseminated infections (n=1) (Tan et al., 2008). The clinicoepidemiological profile of patients diagnosed with keratomycoses of *M. phaseolina* is tabulated below (**Table 1**). *M. phaseolina* is of significant prominence among Deuteromycotina. The bacterium belongs to the phylum Ascomycota, and the Botryosphaeriaceae family (Srinivasan et al., 2009). The genus Macrophomina, originally identified by Petrak in 1923. It is a rare and sparsely distributed genus, that comprises only six species: *M. limbalis, M. phaseoli, M. phaseolina, M. philippinensis, M. pseudoverniae*, and *M. pseudophasaseolina* (Kaur et al., 2012). *M. phaseolina* mainly infects people with diabetes or people with immunocompromised conditions (Srinivasan et al., 2009). To date, only ten documented cases of *M. phaseolina* keratitis have been reported, primarily in tropical regions near the equator (**Table 1**). Like most fungal keratitis, *M. phaseolina* keratitis is often preceded by a history of corneal trauma caused by vegetative matter, although some cases lack an apparent history of trauma (**Figure 4**).

**Table 1:** Global review of cases of keratitis due to *M. phaseolina*.

Year/ case number		Country	Age	Gender	Risk factors	Antifungal therapy	Therapeutic keratoplasty	Outcomes	References
6 cases in a retrospective review (2020-2021)	1	India	46	Male	Foreign body	Natamycin 5%, Ketaconazole 200 mg, Voriconazole 1%	No	Healed scar	Ahirwar et al (Ahirwar et al., 2022)
	2	India	58	Male	Unknown	Natamycin 5%, Ketaconazole 200 mg	Yes	Recurrence after TPK	Ahirwar et al (Ahirwar et al., 2022)
	3	India	65	Male	Vegetative trauma	Natamycin 5%, Ketaconazole 200 mg	Yes	Recurrence after TPK	Ahirwar et al (Ahirwar et al., 2022)
	4	India	45	Female	Unknown	Natamycin 5%, Ketaconazole 200 mg	Yes	No recurrence after TPK	Ahirwar et al (Ahirwar et al., 2022)
	5	India	52	Female	Unknown	Natamycin 5%, Ketaconazole 200 mg	Yes	No recurrence after TPK	Ahirwar et al (Ahirwar et al., 2022)
	6	India	56	Female	Foreign body	Natamycin 5%, Ketaconazole 200 mg	Yes	No recurrence after TPK	Ahirwar et al (Ahirwar et al., 2022)
2 cases in a retrospective review (2007)	7	India	NA	NA	No	topical Amphotericin B 0.15%	No	Healed ulcer	Bagyalakshm et al (Bagyalakshmi et al., 2008)
	8	India	NA	NA	No	topical Amphotericin B 0.15%	No	Healed ulcer	Bagyalakshm et al (Bagyalakshmi et al., 2008)
2012	9	India	70	Female	Vegetative trauma	Natamycin 5%, Voriconazole 200 mg	No	Healed ulcer	Premamalini et al (Premamalini et al., 2012)
2025	10	India	50	Female	Vegetative trauma	Natamycin 5%, Voriconazole 1%, topical Amphotericin B 0.5%, ketoconazole 200mg	No	Healed scar	Tandra et al (Tandra et al., 2025)

NA, Not Available.

#### 3.1.2 Global literature review: *Colletotrichum* spp. keratitis from 1970 to August 1, 2025

A systematic search for FK cases caused by *Colletotrichum* spp. from 1970 to August 1, 2025, was carried out on PubMed, Embase, and Medline, using the keywords ‘Colletotrichum’, ‘Case Reports’, and ‘Infections’. Additionally, we performed a manual search of the reference lists of included studies using a snowball technique to identify further relevant articles. A total of 50 publications on *Colletotrichum* spp infection were ultimately included, 43 describing corneal infections. The search revealed 95 cases of all infections reported by *Colletotrichum* spp. in the world in the last five decades or so. Of these, 72 cases were associated with KF. Other clinical manifestations included endophthalmitis (n=20) (Marangon et al., 2004; Shivaprakash et al., 2011a; Dave et al., 2019; Paniz-Mondolfi et al., 2021; Gunasekaran et al., 2022) and soft tissue infections (n = 3) (O’Quinn et al., 2001; Figtree et al., 2012). The clinicoepidemiological profile of patients diagnosed with *Colletotrichum* spp. keratomycoses is tabulated below (**Table 2**).

**Table 2:** Global review of cases of keratitis due to *Colletotrichum* spp.

Organism	Country	Age	Gender	Risk factors	Diagnosis method	Antifungal therapy	Therapeutic keratoplasty	Outcomes	References
*C. asianum*	India	34	Male	Vegetative trauma	DNA sequencing	Natamycin 5%, Voriconazole 1%	No	Healed scar	Ghorpade et al (Ghorpade et al., 2022)
	India	60	Male	Vegetative trauma	DNA sequencing	Natamycin 5%, Voriconazole 1%	No	Healed scar	Ghorpade et al (Ghorpade et al., 2022)
*C. Glomerella cingulata (C. gloeosporioides)*	India	59	Male	Foreign body	Culture	Miconazole 2%, Amphotericin B 1%	No	Lost to follow up	Shukla et al (Shukla et al., 1983)
*C. coccodes*	India	45	Female	No	Culture	Natamycin 5%	No	Lost to follow up	Natarajan et al (Natarajan et al., 2013)
	India	54	Male	No	Culture	Amphotericin B	No	No recurrence	Kotwal et al (Kotwal et al., 2015)
	Iran	56	Male	Vegetative trauma	IVCM	Natamycin	No	Healed scar	Tabatabaei et al (Tabatabaei et al., 2018)
*C. crassipes*	India	45	Male	Foreign body	Culture	Natamycin 5%	No	Healed scar	Natarajan et al (Natarajan et al., 2013)
*C. dematium*	USA	59	Male	Foreign body	Culture	Natamycin 5%, Voriconazole 1%	No	Healed scar	Giaconi et al (Giaconi et al., 2006)
	India	42	Male	Foreign body	Culture	Natamycin5%, Amphotericin B, Fluconazole 150 mg	No	Lost to follow up	Wankhade et al (Wankhade et al., 2021)
	Czech Republic	56	Female	Foreign body	PCR	Voriconazole 200mg, Itraconazole 100mg, Amphotericin B 0.15%	Yes	No recurrence after TPK	Buchta et al (Buchta et al., 2019)
	India	27	Male	Vegetative trauma	Culture	Natamycin 5%	No	Lost to follow up	Mendiratta et al (Mendiratta et al., 2005)
	India	21	Male	Foreign body	Culture	Natamycin 5%	Yes	Lost to follow up	Natarajan et al (Natarajan et al., 2013)
	India	25	Female	NA	Culture	Natamycin 5%, ketoconazole 200mg	No	Healed scar	Joseph et al (Joseph et al., 2004)
*C. dematium*	India	47	Male	No	Culture	Natamycin 5%	Yes	No recurrence after TPK	Kaliamurthy et al (Kaliamurthy et al., 2004)
	India	19	Male	Foreign body	Culture	Natamycin 5%	Yes	No recurrence after TPK	Kaliamurthy et al (Kaliamurthy et al., 2004)
	India	30	Female	Foreign body	Culture	Natamycin 5%	Yes	No recurrence after TPK	Kaliamurthy et al (Kaliamurthy et al., 2004)
	India	41	Male	No	Culture	Natamycin 5%	Yes	No recurrence after TPK	Kaliamurthy et al (Kaliamurthy et al., 2004)
	India	50	Female	No	Culture	Natamycin 5%	Yes	No recurrence after TPK	Kaliamurthy et al (Kaliamurthy et al., 2004)
	USA	40	Male	Foreign body	Culture	Natamycin 5%	No	Healed ulcer	Fernandez et al (Fernandez et al., 2002)
	USA	28	Male	Vegetative trauma	Culture	Natamycin 5%	No	Healed ulcer	Fernandez et al (Fernandez et al., 2002)
	China	NA	NA	Injury	Culture	Amphotericin B	NA	Healed scar	Liao et al (Liao et al., 1983)
*C. fructicola* *(C. gloeosporioides)*	Taiwan	79	Female	Foreign body	PCR	Amphotericin B, Voriconazole	No	Healed scar	Hung et al (Hung et al., 2020)
	Taiwan	77	Female	NA	PCR	Amphotericin B, Natamycin	No	Healed scar	Hung et al (Hung et al., 2020)
	China	55	Male	Vegetative trauma	NGS	Voriconazole 1%	Yes	No recurrence after TPK	Wang et al (Wang et al., 2024)
	China	59	Female	Vegetative trauma, diabetes	NGS	Voriconazole 1%	Yes	Evisceration	Wang et al (Wang et al., 2024)
*C. fructicola* *(C. gloeosporioides)*	China	54	Male	Vegetative trauma, diabetes	NGS	Voriconazole 1%	Yes	No recurrence after TPK	Wang et al (Wang et al., 2024)
*C. fusiforme* *(C. truncatum)*	Taiwan	76	Male	No	PCR	Fluconazole, Voriconazole	No	Evisceration	Hung et al (Hung et al., 2020)
*C. gloeosporioides*	China	52	Male	Vegetative trauma	DNA sequencing	Voriconazole, Amphotericin B 0.15%, Voriconazole 400mg	No	Healed scar	Wang et al (Wang et al., 2020)
	Spain	74	Male	Vegetative trauma	DNA sequencing	Voriconazole 1%, Voriconazole 200mg	Yes	NA	David et al (Navalpotro Rodríguez et al., 2014)
	Iran	69	Male	No	DNA sequencing	Voriconazole 1%	No	Healed scar	Izadi et al (Izadi et al., 2021)
	Japan	81	Female	Vegetative trauma	Culture	Voriconazole 1%, Natamycin 5%, Voriconazole 400mg	No	Healed ulcer	Shiraishi et al (Shiraishi et al., 2011)
	Japan	71	Female	Vegetative trauma	Culture	Voriconazole 1%, Natamycin 5%	No	Healed ulcer	Shiraishi et al (Shiraishi et al., 2011)
	Japan	60	Male	Vegetative trauma	Culture	Voriconazole 1%, Natamycin 5%, miconazole 0.1%	No	Healed ulcer	Shiraishi et al (Shiraishi et al., 2011)
	Japan	82	Male	Foreign body	Culture	Fluconazole 0.2%, Natamycin 5%, Fluconazole 200mg	No	Healed ulcer	Yamamoto et al (Yamamoto et al., 2001)
	Japan	80	Female	Foreign body	Culture	Voriconazole 1%, Natamycin 5%, Voriconazole 400mg	No	Healed ulcer	Mitani et al (Mitani et al., 2009)
	Japan	81	Female	Vegetative trauma	Culture	Voriconazole 1%, Natamycin 5%,	No	Healed ulcer	Takezawa et al (Takezawa et al., 2010)
*C. gloeosporioides*	Spain	45	Male	Vegetative trauma	Culture	Voriconazole 1%, Voriconazole 400mg	No	Lost to follow up	Breval et al (Zakariya-Yousef Breval et al., 2019)
	Spain	75	Male	Vegetative trauma	Culture	Voriconazole, Voriconazole 400mg	Yes	Recurrence after TPK	María et al (Borrás-Máñez et al., 2016)
	India	45	Male	Foreign body	PCR	Amphotericin B 0.15%, Natamycin 5%, Voriconazole 400mg	No	Healed ulcer	Pote et al (Pote et al., 2017)
	Japan	73	Female	Corticosteroids	Culture	Voriconazole 1%, Natamycin 5%, Voriconazole 400mg	Yes	No recurrence after TPK	Imai et al (Imai et al., 2022)
	Spain	56	Female	Vegetative trauma	Culture	Voriconazole 1%, Amphotericin B 0.05%, Voriconazole 400mg	Yes	No recurrence after TPK	Lamarca et al (Lamarca et al., 2016)
	USA	68	Male	No	Culture	Natamycin 5%	No	Healed scar	Fernandez et al (Fernandez et al., 2002)
	USA	78	Male	No	Culture	Amphotericin B	Yes	No recurrence after TPK	Fernandez et al (Fernandez et al., 2002)
	USA	74	Male	No	Culture	Natamycin 5%, Amphotericin B 0.15%, Itraconazole 100mg	No	No recurrence	Fernandez et al (Fernandez et al., 2002)
	China	46	Male	NA	NGS	Voriconazole 1%	Yes	No recurrence after TPK	Wang et al (Wang et al., 2024)
	China	57	Male	Foreign body	NGS	Voriconazole 1%	Yes	No recurrence after TPK	Wang et al (Wang et al., 2024)
	China	77	Male	Vegetative trauma	NGS	Voriconazole 1%	Yes	No recurrence after TPK	Wang et al (Wang et al., 2024)
*C. graminicola*	USA	27	Male	No	Culture	Natamycin 5%, Amphotericin B, Fluconazole	Yes	No recurrence after TPK	Ritterband et al (Ritterband et al., 1997)
	India	44	Male	No	Culture	Natamycin 5%, Fluconazole 0.3%, Ketoconazole 400 mg	No	Healed ulcer	Yegneswaran et al (Yegneswaran et al., 2010)
*C. tropicale* *(C. gloeosporioides)*	Taiwan	72	Male	Foreign body	PCR	Natamycin	No	Healed scar	Hung et al (Hung et al., 2020)
	Taiwan	63	Female	Vegetative trauma	PCR	No	No	Healed scar	Hung et al (Hung et al., 2020)
	Taiwan	22	Male	Foreign body	PCR	No	No	Healed scar	Hung et al (Hung et al., 2020)
*C. truncatum*	Cuba	41	Male	No	DNA sequencing	Miconazole 1%, Ketoconazole 1%, Natamycin 5%, Ketoconazole 200mg	Yes	No recurrence after TPK	Llamos et al (Llamos et al., 2016)
	Cuba	70	Male	No	DNA sequencing	Miconazole 1%, Natamycin 5%	Yes	No recurrence after TPK	Llamos et al (Llamos et al., 2016)
	India	60	Female	Vegetative trauma	PCR	Oral and topical fluconazole	Yes	Phthisis bulbi	Shivaprakash et al (Shivaprakash et al., 2011b)
	India	38	Male	Corticosteroids	PCR	Amphotericin B, Itraconazole	No	No recurrence	Shivaprakash et al (Shivaprakash et al., 2011b)
	India	70	Male	No	PCR	No	Yes	No recurrence after TPK	Shivaprakash et al (Shivaprakash et al., 2011b)
	India	58	Male	Foreign body	PCR	No	Yes	Corneal opacity	Shivaprakash et al (Shivaprakash et al., 2011b)
	India	50	Male	Vegetative trauma	PCR	Amphotericin B	No	No recurrence	Shivaprakash et al (Shivaprakash et al., 2011b)
	Jamaica	87	Male	No	DNA sequencing	Amphotericin B	Yes	Recurrence after TPK	Squissato et al (Squissato et al., 2015)
*C. truncatum*	India	12	Male	Vegetative trauma	DNA sequencing	Itraconazole 1%, Natamycin 5%	No	Healed scar	Christy et al (Christy et al., 2020)
	India	60	Female	Vegetative trauma	DNA sequencing	Natamycin 5%, Oral and topical fluconazole	No	Lost to follow up	Ghatole et al (Ghatole et al., 2014)
	Taiwan	62	Male	Vegetative trauma	PCR	No	No	Evisceration	Hung et al (Hung et al., 2020)
	China	54	Female	Vegetative trauma	NGS	Voriconazole 1%	Yes	No recurrence after TPK	Wang et al (Wang et al., 2024)
	China	51	Female	Vegetative trauma	NGS	Voriconazole 1%	Yes	No recurrence after TPK	Wang et al (Wang et al., 2024)
	China	66	Female	Vegetative trauma	NGS	Voriconazole 1%	Yes	No recurrence after TPK	Wang et al (Wang et al., 2024)
*Colletotrichum* spp	India	80	Female	No	Culture	Natamycin 5%	Yes	No recurrence after TPK	Kaliamurthy et al (Kaliamurthy et al., 2004)
	Spain	23	Male	Vegetative trauma	Culture	Amphotericin B 0.5%	No	Healed scar	Guardiola et al (Morcillo Guardiola et al., 2014)
	USA	34	Male	Trauma	Culture	NA	NA	NA	Fernandez et al (Fernandez et al., 2002)
	USA	78	Female	Corneal erosion	Culture	Natamycin 5%, Amphotericin B 0.15%	No	Healed scar	Fernandez et al (Fernandez et al., 2002)
	USA	35	Male	Corticosteroids, Foreign body	Culture	Natamycin 5%	No	Healed ulcer	Fernandez et al (Fernandez et al., 2002)
	USA	69	Male	Corticosteroids, Vegetative trauma	Culture	Natamycin 5%	No	Healed scar	Fernandez et al (Fernandez et al., 2002)

NA, Not Available; *(C. gloeosporioides)* = Species within the complex of *C. gloeosporioides* complex; *(C. truncatum)* = Members within the complex of *C. truncatum.*

*Colletotrichum* spp. are classified under the Deuteromycotina subdivision of fungi. The fungus belongs to the class Coelomycetes, order Melanconiales, and the family Melanconiaceae (Wenping and Zhiming, 1994). To date, there are 11 recognized anthracnose complexes (Jayawardena, 2016). In 2019, Damm et al. reported three additional anthracnose complexes (Damm et al., 2019). Therefore, there are a total of 14 complexes in the genus *Colletotrichum* spp., namely *C. acutatum* complex, *C. boninense* complex, *C. caudatum* complex, *C. dematium* complex, *C. destructivum* complex, *C. dracaenophilum* complex, *C. gigasporum* complex, *C. gloeosporioides* complex, *C. graminicola* complex, *C. magnum* complex, *C. orbiculare* complex, *C. orchidearum* complex, *C.* sp*aethianum* complex, and *C. truncatum* complex. *Colletotrichum* spp. exhibits a wide geographic distribution, being especially prevalent in tropical and subtropical regions. It is ranked as the eighth most phytopathogenic fungus in the world (Dean et al., 2012). To date, 73 cases of *Colletotrichum* spp. keratitis have been documented, including the present case, which reports on *C. fructicola* keratitis, a member of the *C. gloeosporioides* complex.

**Figure 5:**
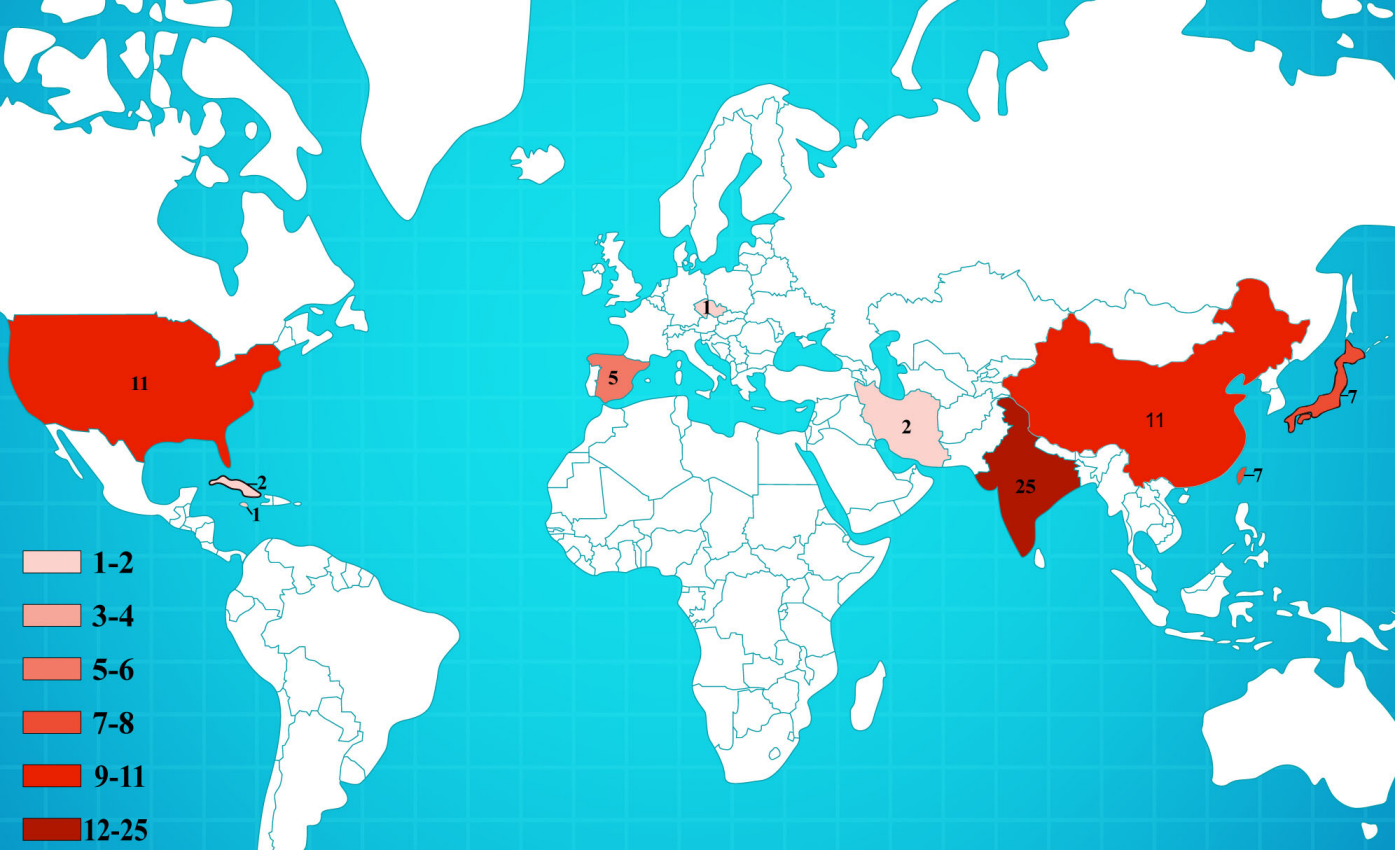
Global distribution of reported case reports of keratitis caused by *Colletotrichum* spp.

**Figure 6:**
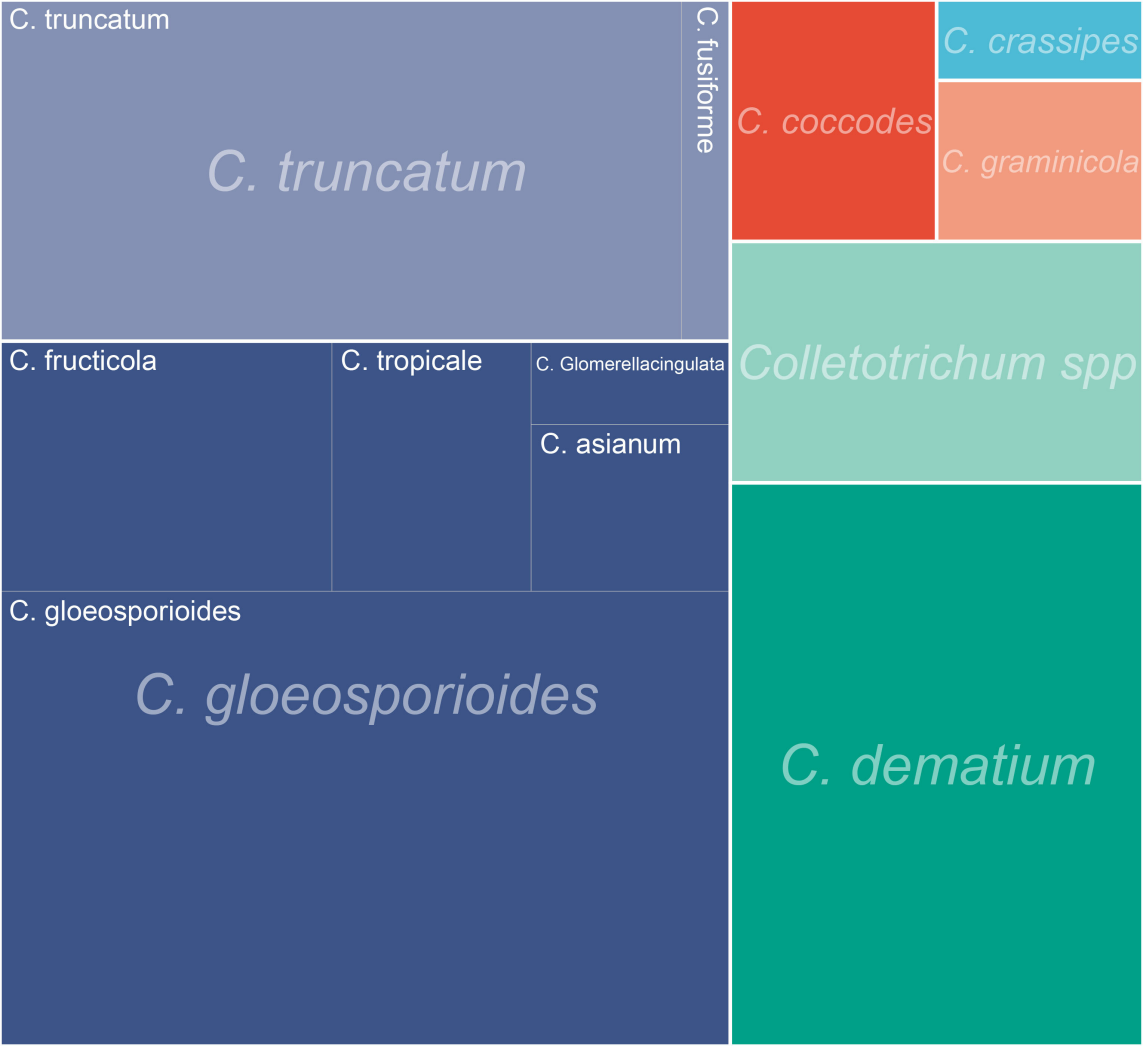
The fungal category of *Colletotrichum* spp causing corneal infection.

**Figure 7:**
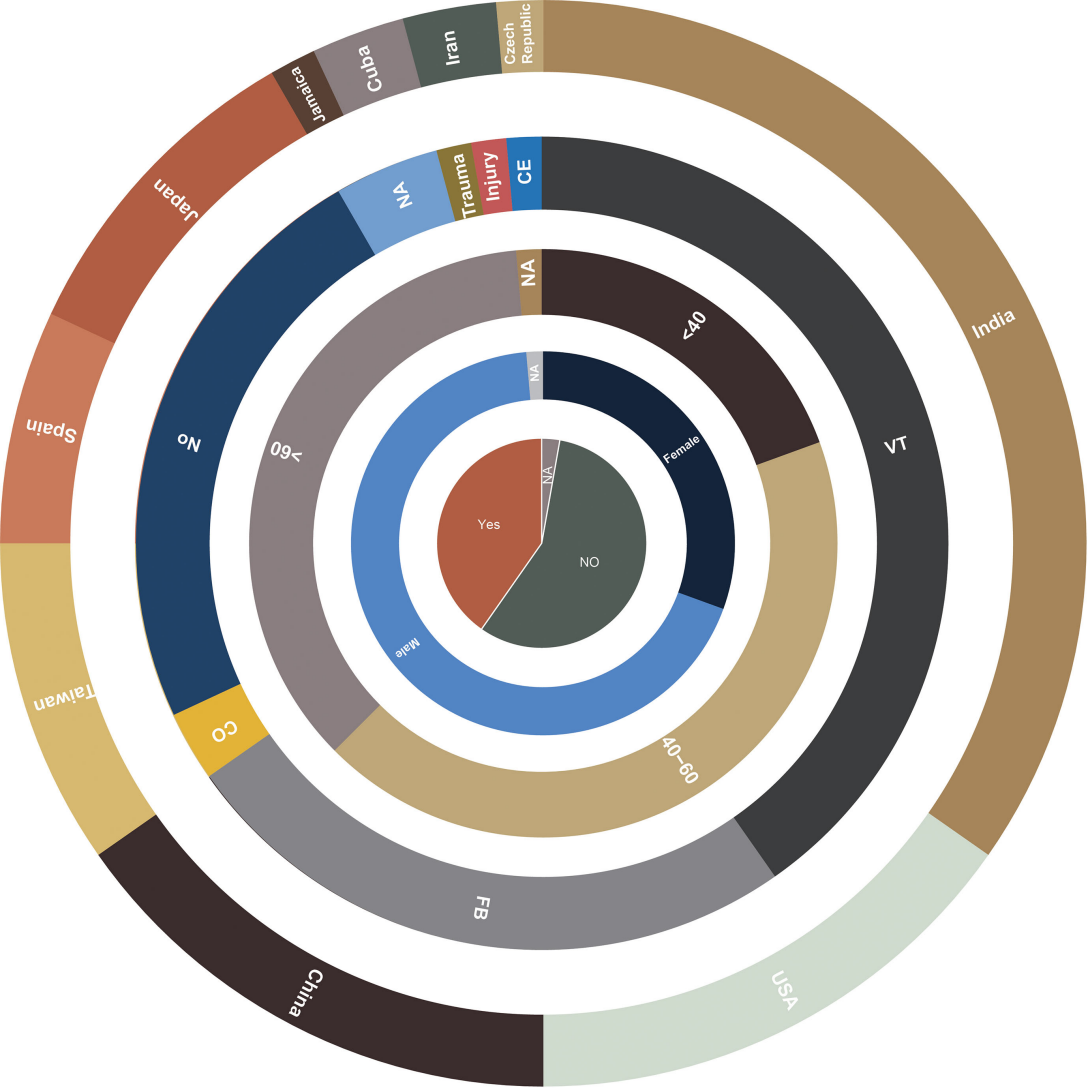
Statistical chart depicting the clinical and epidemiological characteristics of corneal infections caused by *Colletotrichum* spp. Country, risk factors, age, sex, and therapeutic keratoplasty are shown in order from the outer ring to the inner ring. (NA=not available, FB=foreign body, VT=vegetative trauma, CO=corticosteroids, CE= corneal erosion).

As observed in other cases of fungal keratitis, *Colletotrichum* spp. is predominantly reported in tropical regions close to the equator (**Figure 5**) (Brown et al., 2021). However, there have been documented cases in temperate areas, such as the Czech Republic and Iran. The primary etiological agents of fungal keratitis in *Colletotrichum* spp. are *C. gloeosporioides, C. dematium*, and *C. truncatum* (**Table 2**) (**Figure 6**). The incidence rate of *Colletotrichum* spp. keratitis is markedly higher among men (49/72 = 68.06%). There is a significant correlation between this infection and a history of plant-related trauma, a common precursor of fungal keratitis (**Figure 7**).

Cases of corneal infections caused by the two types of plant pathogens are primarily concentrated in tropical and subtropical countries such as India and China, which may reflect the unique epidemiological background and the spectrum of infectious diseases, but publication or reporting bias cannot be ruled out.

### 3.2 Challenges in clinical and microbiological diagnosis

The study indicates that the risk factors and clinical manifestations of keratitis caused by the two aforementioned plant pathogenic fungal infections are similar to those associated with other fungal infections. Both patients showed mycelial placentation, feathery margins, and gray infiltration in our article. Before coming to our facility, they had received treatment at other hospitals, including antibiotics, traditional eye medications, and steroids or NSAIDs. These treatments were administered due to the inability to identify the pathogen, resulting in masked clinical symptoms and delayed disease management. There are no distinct ocular indicators that differentiate the rare fungal keratitis induced by the two pathogenic plant fungi in question from other forms of fungal keratitis. The clinical presentations of fungal keratitis overlap significantly, and due to the various susceptibilities of various fungi to antifungal agents, accurate identification of the specific fungal group is crucial for customized treatment strategies. Recognizing infrequently identified fungi can facilitate a comprehensive understanding of disease prevalence and improve the prognosis and management of rare cases of fungal keratitis.

Fungal culture remains the gold standard for diagnosing fungal keratitis (Brown et al., 2021). However, this process is time-consuming and lacks sensitivity. The fungal cultures of both patients discussed in this report were negative. Other commonly used clinical diagnostic methods are inadequate for the early detection of fungal keratitis. In both reports, definitive diagnoses were achieved using mNGS, a novel technique for pathogen detection. The emergence of newer technologies highlights the inadequacies of older methods, which do not provide accurate and timely diagnoses necessary to guide treatment and preserve the vision of patients. This insufficiency delays effective treatment and has serious consequences for patients. mNGS can overcome the drawbacks of conventional detection techniques when dealing with keratitis caused by rare or unusual pathogens. Not only does it effectively detect pathogens in the early stages of infection, but it also provides critical evidence for the improvement of real-time clinical treatment plans by dynamically monitoring changes in the pathogen population (Zhao et al., 2025).

### 3.3 Challenges in clinical treatment

The management of fungal keratitis remains particularly challenging. Current antifungal agents are limited by poor ocular penetration and low bioavailability, often necessitating prolonged treatment courses that frequently produce unsatisfactory results. In addition, delays in clinical diagnosis, long intervals for pathogen identification, and the time-consuming process of antifungal susceptibility testing further exacerbate these challenges, contributing to poorer prognoses (Sharma et al., 2022).

In clinical practice, empirical treatment with broad-spectrum topical antibiotics is commonly initiated in cases of keratitis with unclear etiology (McDonald et al., 2014). Although this approach is intended to provide immediate coverage, it may inadvertently disrupt the ocular microbiome and alter antimicrobial susceptibility profiles, increasing the risk of selecting drug-resistant pathogens (Odonkor and Addo, 2011). The development of antifungal resistance during treatment is an issue. A subgroup analysis of the Mycotic Ulcer Treatment Trial I (MUTT I) revealed that patients with IK treated with topical antifungal medications experienced a mean increase in minimum inhibitory concentration (MIC) of 2.14 times per year, even after adjusting for pathogen factors (Prajna et al., 2016). Antimicrobial resistance (AMR) has become a critical global health concern in the 21st century, largely driven by inappropriate or empirical antimicrobial use in the context of diagnostic uncertainty. In this evolving ‘post-antibiotic’ era, judicious selection of antimicrobial agents is imperative (Alanis, 2005). Therefore, the early application of advanced diagnostic technologies, such as mNGS, holds particular promise in facilitating accurate and timely identification of pathogens. Such strategies are important to direct targeted antimicrobial therapy, reduce resistance development, and ultimately protect patient visual outcomes. Although the two cases in this study did not receive standardized treatment, they had good treatment outcomes once medication modifications were made according to the diagnosis of mNGS.

## 4 Conclusion

Fungal keratitis is a prevalent and severe infectious eye disease characterized by grave progression and poor treatment results, often leading to devastating consequences. Early diagnosis of fungal keratitis is crucial for effective treatment and restoration of the patient’s vision. The management of fungal keratitis is challenging. Early diagnosis and species identification pose significant challenges, even for clinically experienced specialists. Traditional diagnostic methods, including fungal culture and microscopic examination, often do not provide accurate diagnoses and precise species identification during the initial stages of a disease. On the contrary, mNGS produces more reliable results for early diagnosis, which is crucial to guide the appropriate treatment. However, conventional microbiological methods remain indispensable for diagnosing infectious keratitis. Despite the significant diagnostic advantages of mNGS, its high cost and restricted availability in some areas may limit its normal use in clinical practice.
